# Effect of Oral Anticoagulant Therapy on Coagulation Activity and Inflammatory Markers in Patients with Atrial Fibrillation Undergoing Ablation: A Randomized Comparison between Dabigatran and Warfarin 

**Published:** 2013

**Authors:** Shahideh Amini, Kheirollah Gholami, Hooman Bakhshandeh, Bahram Fariborz Farsad

**Affiliations:** a *Clinical Pharmacy Department, Pharmacy School, Tehran University of Medical Sciences, Tehran, Iran.*; b *Faculty of Pharmacy and Research Center for Rational Use of Drugs, Tehran University of Medical Sciences, Tehran, Iran.*; c *Cardiac Intervention Research Center, Rajaie Cardiovascular Medical and Research Center, Tehran University of Medical Sciences, Tehran, Iran.*; d *Rajaie Cardiovascular Medical and Research Center, Tehran University of Medical Sciences,Tehran,Iran. *

**Keywords:** Anticoagulant therapy, Inflammatory, Dabigatran, Warfarin

## Abstract

Atrial fibrillation (AF) is associated with inflammatory and hypercoagulability state. Previous studies evaluated the safety and efficacy of dabigatran and warfarin in prevention of thrombothic complications. This study was intended to assess the influence of these drugs on hemostatic and inflammatory markers among patient underwent pulmonary vein ablation. A total of 100 patients with AF who underwent catheter ablation were randomized to treatment with dabigatran (D) 110 mg twice daily or warfarin (W) adjusted to an international normalized ratio (INR) of 2.0 to 3.0 for 3 months after ablation procedure. C - reactive protein (CRP), D-dimer, prothrombin fragment F1 + 2 (F1 + 2), were measured at baseline before ablation procedures, after 30 days and after 90 days of treatment. After 3 months, the D-dimer was 164.9 ± 48.9 in Dabigatran and 197.2 ± 58.6 in warfarin group, F1 + 2 was 0.4 ± 0.2 in dabigatran and 0.8 ± 0.2 in warfarin group and CRP level was 1.8 ± 1.6 in Dabigatran and 5.1 ± 5 in warfarin group. (All p-values < 0.05) The results showed that treatment with dabigatran made greater changes in the serum level of CRP, D-dimer, F1 + 2. The pattern of changes in serum CRP levels D-dimer, F1 + 2 is much faster and with a greater slope in the dabigatran group.

## Introduction

Atrial fibrillation (AF) remains the most common cardiac arrhythmia which is associated with increased morbidity and mortality. Thromboembolic events are the most prevalent complication of AF (1, 2). 

The underlying patho-physiologies of AF are multifactorial. Recent studies have indicated that atrial fibrillation; is aheterogeneous disease with numerous interacting mechanisms involved in the genesis, maintenance and persistence of it (3, 4). 

Current evidences have suggested that coagulation activation, abnormal inflammation and endothelial dysfunction play an important role in the pathogenesis of AF (5, 6). Elevation of inflammatory markers such as interleukin-6 and C-reactive protein were seen in AF patients (7, 8). Indeed hypercoagulable or prothrombotic states may be detected in AF patients and there is a significant association between inflammatory markers and thrombogenesis, in which elevation of inflammatory markers leads to an increase in the risk of vascular events (8-10). 

Despite oral anticoagulation (OAC), AF patients continue to suffer from thromboembolic stroke and in many cases the occurrence of cerebral infarction is silent (11). Until recent years warfarin has been the only effective oral anticoagulant therapy for the prophylaxis of stroke in AF patients. Nevertheless, the Randomized Evaluation of Long-Term Anticoagulation Therapy (RE-LY) study showed superiority of dabigatran over warfarin in the prevention of stroke and systemic embolism (12).

While dabigatran has a place in the therapeutic prevention stroke and systemic embolism associated with AF, there is no study that has compared the effect of this drug on inflammatory and hemostatic markers with warfarin, so we performed a randomized trial to compare the effect of dabigatran at doses of 110 mg twice daily with warfarin adjusted to an international normalized ratio (INR) of 2.0 to 3.0 in AF patients post single circumferential pulmonary vein ablation on markers of thrombin generation (prothrombin fragment F1 + 2 (F1 + 2)), fibrin turnover (fibrin D-dimer) and inflammation (c-reactive protein, CRP).

## Experimental

*Patients*

We performed a, randomized controlled trial of patients undergoing AF ablation for drug-refractory, symptomatic AF between January 2010 and July 2011 at Rajaie Cardiovascular Medical and Research center in Tehran, a tertiary health care providing hospital. The study protocol was approved by the local institutional review board and informed written consent was obtained from all the participants after registration in the study. 

Patients with drug-refractory, symptomatic AF who had indications for ablation included in this study. Patients were excluded in the presence of a severe heart-valve disorder, stroke within 14 days or severe stroke within 6 months before screening, a condition that increased the risk of hemorrhage, a creatinine clearance of less than 30 mL per minute, an active liver disease, and pregnancy at the time of the ablation procedure. Registration was performed by an electrophysiologist who was a member of scientific board of our institute.

*Randomization, study groups and endpoints*

After providing AF ablation procedures and participants registration, 100 patients were randomly assigned to receive 110 mg dabigatran (Pradaxa^®^, BoehringerIngelheim) twice daily, or to receive warfarin adjusted to an international normalized ratio (INR) of 2.0 to 3.0, for at least 90 days after the AF ablation procedure. 

Randomization was performed using the balanced block method (block of four). Randomization concealment was applied using the sealed envelope method. The process of randomization and concealment was carried out by one of the authors who did not participate in the patients’ enrollment, treatment, follow up and data collection.

Primary endpoints of the study were to determine and compare the changes in blood levels of F1+2, D-dimer and CRP between two study groups. Comparison of the thrombotic complications and safety profile was the secondary endpoints of the study.

*Blood sampling and assays*

Venous blood was drawn with a 21-22 gauge needle. Blood was collected into vacutainer tubes containing citrate 3.8%. The blood was centrifuged within at 2000 g for 20 min and stored at -70 until analysis. All laboratory technicians who were involved in blood sampling, analyzing and reporting the results had been masked to the randomized status and treatment of the study participants. The INR, PT, aPTT, F1 + 2, D-dimer and CRP were measured at baseline before ablation procedures, after 30 days (in the first visit of patients after ablation in our center) and after 90 days of treatment (minimum anti-coagulation therapy after AF ablation (13). In the warfarin group, Time in Therapeutic range (TTR) was calculated by the Rosendaal method (14).

The APTT and PTT were analyzed by C.K PREST^®^and NEOPLASTINE ^®^cl respectively. F1 + 2 and D-dimer were analyzed with commercial immunoassays (Enzygnost^®^, Dade Behring for F1+2 and VIDAS^®^for D-dimer,). Levels of CRP were measured with CRP-Latex Immunoturbidometric Assay (CRP-LIA). All analyses were performed at the laboratory in our institute.

*Statistical analysis*

Data were described as mean ± standard deviation for interval and count (percent) for categorical variables. One sample Kolmogorov-Smirnov test was applied to find the fitness of interval variables with normal distribution. Baseline data were compared between the two study groups with Student’s t test (for interval variables) and Pearson’s chi square or Fisher’s exact tests (for categorical variables). Repeated measure analysis of variance (ANOVA) models were applied to investigate the changes of study outcomes through the time and the associations with the types of treatment (study groups) and also the interactions. Bonferroni post-hoc test was applied for pair wise comparisons. P-value < 0.05 was considered as statistically significant. Intention to treat (ITT) approach was considered for data analysis.

Multivariate analysis was performed by using generalized estimating equations (GEE) method. In these models, the adjusted associations between blood levels of CRP in the baseline, first month and third month after treatment and the type of treatment (dabigatran or warfarin) were investigated. 

IBM^®^ SPSS^®^ Statistics 19 for Windows^®^ (IBM Corporation, New York, USA, 2010) was used for statistical analysis.

## Results

*Study participants and their baseline characteristics*

From June 2011 to July 2012, a total number of 112 patients were assessed for the study inclusion criteria. One hundred people who met the criteria were randomly assigned to the dabigatran or the warfarin group, 50 in each group. The mean age of the participants was 58 ± 11.7 years and the female/male ratio was 52/48. The patients’ characteristics are summarized in Table 1. No significant differences were observed between the study groups.

**Figure 1:**
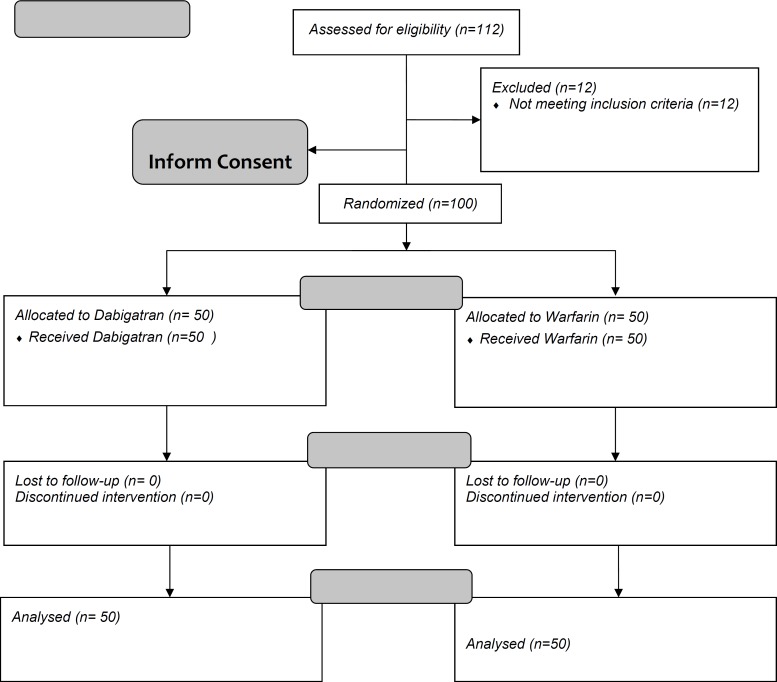
Flow Diagram of the Study Participants

All the participants received the assigned treatment and were followed until the three months after. No withdrawal or loss to follow up occurred after treatment (Figure 1). The INR level was in therapeutic range in all the patients. The mean percent time in therapeutic INR range (TTR) in this group at the third month after treatment was 52% ± 6.8%.

**Table 1:** Baseline characteristics in the two study groups

**Treatment Group**			**p-value**
**Dabigatran** **(n = 50)**	Dabigatran (n = 50)	**Warfarin** **(n = 50)**	
**Age years**	56 ± 14.4	60 ± 8.2	0.442
**Age ≥ 65 years**	18 (36%)	11 (22%)	0.123
**Sex (F/M)**	25/25	27/23	0.689
**Body Mass Index**	26.4 ± 2.9	28.7 ± 4.1	0.161
**AF Duration months**	25.6 ± 25.5	25.7 ± 16.9	0.998
**Heart Failure (n = 11)**	6 (12%)	5 (10%)	0.749
**Hypertension (n = 51)**	28 (56%)	23 (46%)	0.317
**Diabetes (n = 30)**	17 (34%)	13 (26%)	0.180
**Coronary Artery Disease (n = 18)**	7 (14%)	11 (22%)	0.298
**Chronic renal insufficiency (n = 6)**	5 (10%)	1 (2%)	0.204
**Alcohol Drinking (n = 13)**	5 (10%)	7 (14%)	0.538
**Medical Treatments**			
**Aspirin (n = 22)**	10 (20%)	12 (24%)	0.273
**ACE Inhibitors (n = 35)**	18 (36%)	17 (34%)	0.834
**Statins (n = 33)**	19 (38%)	22 (44%)	0.542
**Beta Blockers (n = 31)**	12 (24%)	19 (38%)	0.130
**Calcium Channel Blockers (n = 23)**	10 (20%)	13 (26%)	0.476
**CHADS2-VASc score**	1.55 ± 1.44	2 ± 0.87	0.177
**0**	18 (36%)	12 (24%)	
**1**	5 (10%)	11 (22%)	
**≥2**	27 (54%)	28 (56%)	
**HAS-BLED score**	0.69 ± 0.2	0.73 ± 0.24	0.142
**0**	27 (54%)	28 (56%)	
**1**	18 (36%)	17 (34%)	
**≥2**	5 (10%)	5 (10%)	

*Comparison of outcomes and safety between the study groups* *D-dimer*

**Figure 2:**
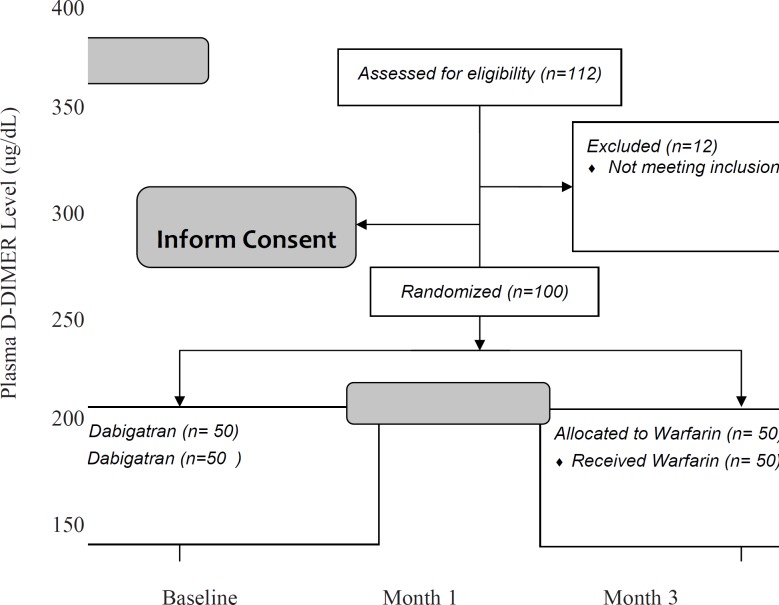
Comparison of changes in blood D-dimer levels between two groups of patients

Results are presented in Table 2. In addition, changes in plasma levels of D-dimer in three months of follow-up and the comparisons between the two study groups are shown in Figure 2. These findings showed that the plasma levels of D-dimer after 3 months were significantly different between the two groups (164.9 ± 48.9 in dabigatran and 197.2 ± 58.6 in the warfarin group, p-value = 0.003). 

**Table 2:** Comparison of outcomes between the study groups

	**Treatment Group**			**P value**
	**Dabigatran** **(n = 50)**	**Warfarin** **(n = 50)**		
**D-dimer**				
**Baseline**	333.6 ± 81.4†, ‡	303.9 ± 118.1†,‡		0.147
**Month 1**	287.9 ± 91.7†, §	262.2 ± 69.3†, §		0.118
**Month 3**	164.9 ± 48.9‡, §	197.2 ± 58.6‡, §		0.003
**Time-Treatment Interaction** *		*0.013*		
**Prothrombin fragment F1+2**				
**Baseline**	1.8 ± 0.6†, ‡	1.6 ± 0.8†,‡		0.161
**Month 1**	1.5 ± 0.8†, §	1.4 ± 0.6†, §		0.450
**Month 3**	0.4 ± 0.2‡, §	0.8 ± 0.2‡, §		<0.001
**Time-Treatment Interaction** *		*0.002*		
**C Reactive Protein**				
**Baseline**	11.3 ± 12.2	7.4 ± 10.7		0.100
**Month 1**	5.9 ± 4.9 †	6.5 ± 7.5		0.625
**Month 3**	1.8±1.6 †	5.1 ± 5.0		<0.001
**Time-Treatment Interaction** *			*0.041*	

* Assessed by repeated measure ANOVA models.

†, ‡, §: Statistically significant results in pairwise comparisons within groups, using Bonferroni post-hoc test.

Repeated measure ANOVA model revealed that the interaction between treatment and study groups was significant (p-value = 0.013). It means that the effect of dabigatran to reduce the blood level of D-dimer was greater than warfarin. Between the first and third months after treatment, the rate of decrease was faster and the final blood levels of D-dimer were the less in dabigatran group (Figure 2).

*Prothrombin fragment F1 + 2*

Table 2 shows that the difference between dabigatran and warfarin groups in the blood levels of F1 + 2 was significant (0.4 ± 0.2 in dabigatran and 0.8±0.2 in warfarin group, p-value < 0.001).

**Figure 3:**
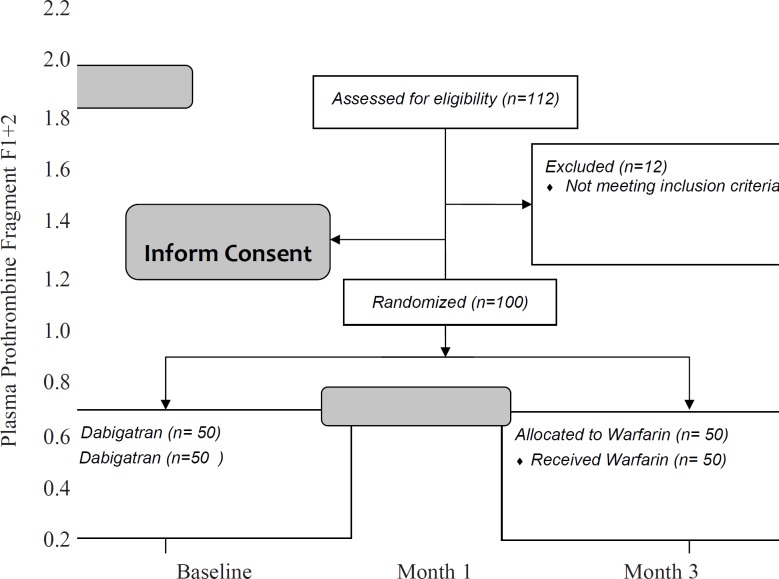
Comparison of changes in Prothrombine Fragment F1+2 levels between two groups of patients.

Statistical analysis confirmed that the interaction between type of treatment and F1+2 was significant (p-value = 0.002). Similar to D-dimer, reduction in blood level of F1+2 was significantly faster in treatment with dabigatran rather than warfarin after the first month (Figure 3). 

*C-reactive protein*

**Figure 4:**
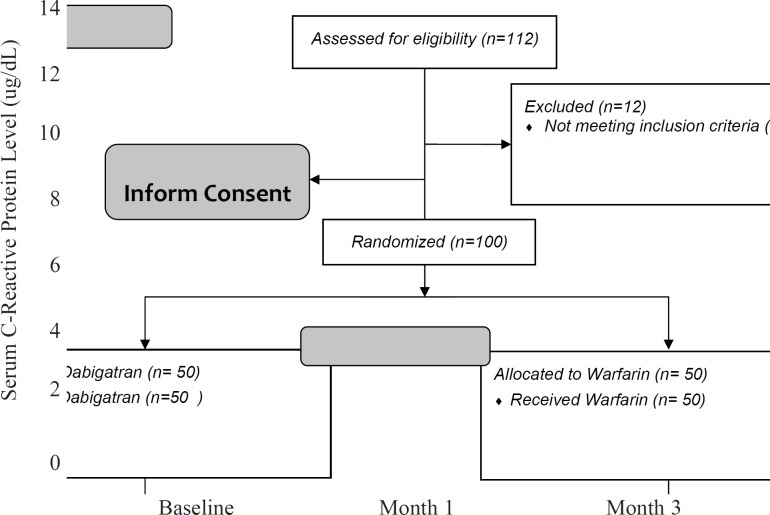
Comparison of changes in serum C - reactive protein between two groups of patients

After 3 months, the CRP level significantly decreased in the dabigatran group (1.8 ± 1.6 in dabigatran and 5.1 ± 5.0 in the warfarin group, p-value < 0.001). Repeated measure ANOVA model suggested that there was a significant interaction between treatment and study groups (p-value = 0.041). It means that similar to D-dimer, the pattern of changes in serum CRP levels is much faster and with a greater slope in the dabigatran group. By other words, in spite of the greater levels of serum CRPin the dabigatran group at the baseline, the drug made a significant decrease in the patients’ serum CRP (Figure 4).

*Multivariate analysis*

Multivariate analysis was performed by GEE model and the result is presented in Table 3. After adjustment for several determinants, it was observed that significant association existed between the type of treatment and changes in CRP levels (*β *= -8.21 ± 2.57, p-value = 0.001). It means that dabigatran caused more reduction in blood CRP levels compared to warfarin. In addition, diabetes showed significant associations after adjustment (Table 3). By other word, among different predictors, only diabetes showed a significant relationship with higher serum CRP levels. Other important factors, such as affliction to heart failure, chronic kidney disease and hypertension didn’t show any statistically significant associations with CRP levels. 

**Table 3:** Multivariate analysis to find the adjusted association between serum CRP and anti-coagulation therapy method, adjusted for other determinants

**Coefficient (β)**	**Coefficient (β)**	**SE for ** ***β***	**P value**
**Treatment (Dabigatran)**	-8.206	2.5739	0.001
**Female sex**	3.800	4.2175	0.368
**Age**	.005	.0511	0.921
**Heart Failure**	-13.885	10.4636	0.185
**Hypertension**	4.094	12.9830	0.752
**Diabetes**	14.603	2.5893	<0.001
**Coronary Artery Disease**	-.736	7.8232	0.925
**Chronic renal insufficiency**	8.504	11.0892	0.443
**Aspirin**	.267	2.7622	0.923
**ACE inhibitors**	-6.393	9.0849	0.482
**Beta blockers**	4.790	6.6190	0.469
**Statins **	-4.494	4.2877	0.481
**Calcium Channel Blockers**	-5.722	5.6481	0.311

*Safety and adverse drug reactions*

No bleeding and any other adverse effects were observed. 

## Discussion

The results of this study show that prescription of dabigatran in patients with AF who undergo catheter ablation leads to the decrease in D-dimer, CRP and F1+2 in a period of three months after ablation and this decrease is higher in comparison to the group who have received warfarin. Although the pathophysiology of atrial fibrillation and the mechanism that causes increase in the risk of thromboembolic events in this disease has not been identified correctly, findings show that inflammatory processes have an important role in the onset of the disease and its continuance and this disease is associated with a hypercoagulable and prothrombotic state (3, 15). Yet it is not clearly obvious whether inflammation is the outcome of the disease or the etiology of the disease (16). Somestudies believe that early recurrence of AF after ablation may be due to inflammatory processes. In a study recently performed, it has been proven that the level of interleukin 6 and CRP are relatively associated with the recurrence rate of AF after ablation (17, 18).

In addition, there is a mutual association between inflammatory processes and the hemostatic system. Inflammation leads to elevation of coagulation factors and decrease of fibrinolytic pathways. These changes result in a hypercoagulable and thrombotic state. A higher level of D-dimer and VWF is associated with an increase in the prevalence of stroke (19, 20).

The level of hemostatic markers may predict the course of the disease (21).

In addition to the prognostic role of hemostatic markers, the impacts of drugs have also been evaluated. Therapeutic doses of warfarin and an INR in the range of 2-3 may decrease the level of markers, while aspirin and low doses of warfarin may have no effect on hemostatic markers (22, 23).

On the other hand, other studies have proved that warfarin has no effect on platelet markers, but it decreases the level of coagulation markers. This finding has been clinically approved that activation of the coagulation cascade has a more determinant role in comparison to the increase in platelet activity and also warfarin is more efficient than aspirin in the prevention of thromboembolic events (6).

Not only hypercoagulable states can occur after inflammatory processes, but also the coagulation system may affect the inflammatory process significantly.

Among these coagulable factors, thrombin, which is considered as an important component and a pivotal central enzyme in the hemostatic system, can increase the expression of specific cell receptors on mononuclear cells or endothelial cells and consequently induced inflammatory cytokine and growth factors production. Thrombin by means of binding to protease-activated receptors (PARs) may stimulate the secretion of proinflammatory mediators and several growth factors (24, 25).

Direct thrombin inhibitor as a therapeutic agent, which may affect thrombin as the last target of the coagulation pathway, may have an important role on the coagulation pathway and probably the inflammatory process. In animal studies, thrombin has increased inflammation and thrombin inhibitor medications were able to decrease the thrombin-induced inflammation (26).

In the animal model, it was determined that dabigatran may decrease fibrosis and inflammation and may also decrease lung injury in the interstitial lung disease model (27).

In the present study, dabigatran has been able to decrease inflammatory and also hemostatic markers, while this decrease was less in the warfarin group. In the literature, no anti inflammatory effect has been detected for warfarin and only hemostatic factors has been influenced by the drug treatment (24, 28, 29).

In clinical studies and trials that compared dabigatran and warfarin, safety and bleeding complications and also the incidence of thrombotic events such as the incidence of stroke have been compared (12).

No comparison has been made between the impacts of these drugs on these markers. Besides, the clinical effect of these changes has not been evaluated till now. Since there is an association between the incidence of disease attacks and the inflammatory processes, the effect of dabigatran on these inflammatory markers may be of importance (30).

It has been observed that in addition to the high risk of accidents such as stroke AF patients face, these patients in spite of anti coagulant therapy are also at risk for microthrombotic events such as dementia. Suppression of D-dimer may decrease the probability of dementia in these patients (31-33).

The effect of dabigatran on thrombin and consequently the decrease of inflammatory markers which have been detected in this study and also the higher decrease of D-dimer levels in comparison to warfarin may enlighten researchers regarding the positive results of this drug on lowering microthrombotic events.

The small sample size and not evaluating the recurrence rate of AF in the patients were the limitations of this study. Therefore, we suggest performing studies in the future to evaluate the clinical impact of change in marker levels. 
